# Supplementary figures and images for: Starch biotransformation into isomaltooligosaccharides using thermostable alpha-glucosidase from Geobacillus stearothermophilus
Source: PeerJ. 2018 Jun 21;6:e5086. doi: 10.7717/peerj.5086 (PMC6015754; doi:10.7717/peerj.5086)

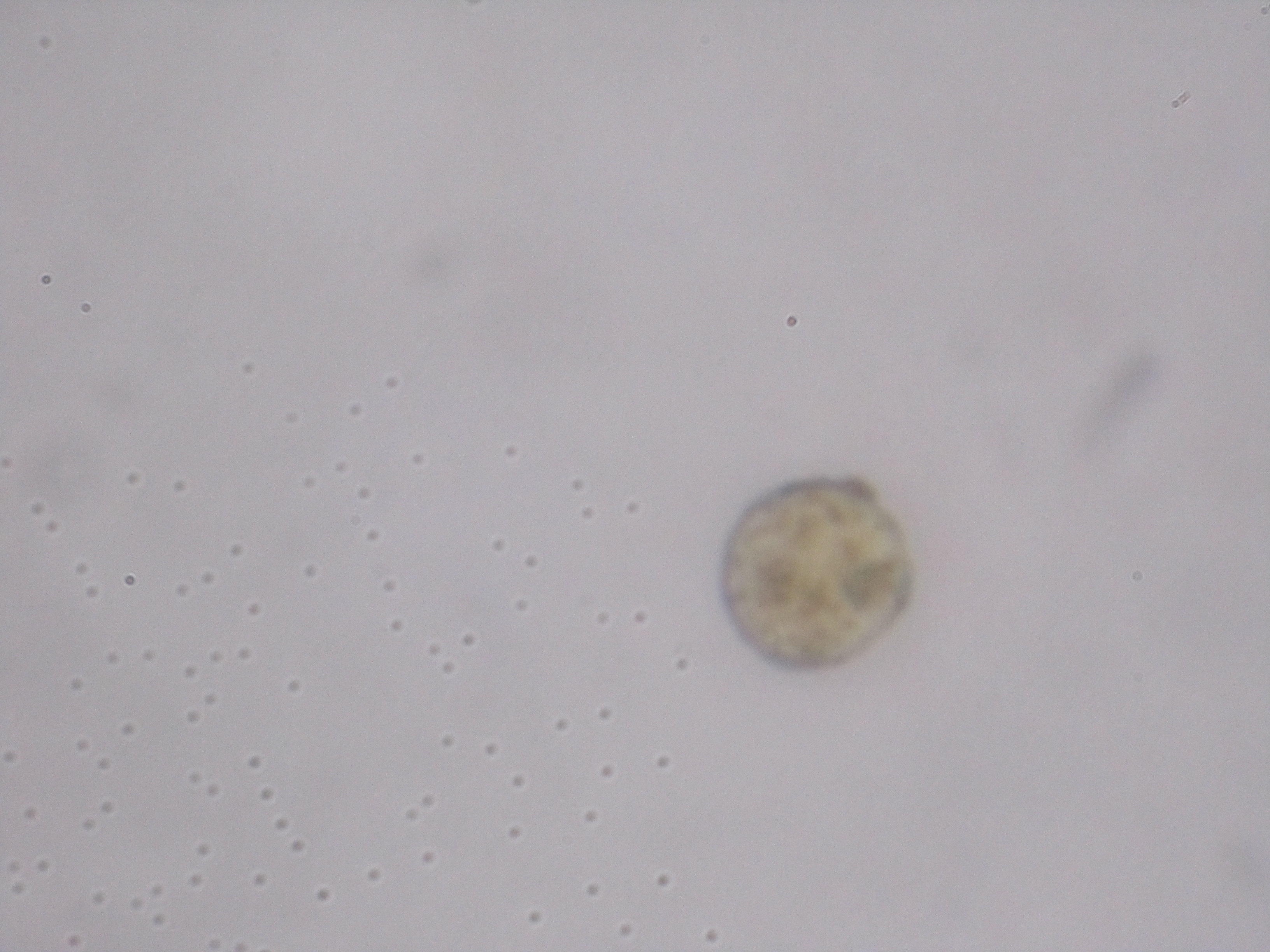

Supplement: Supplemental Information 5 [file peerj-06-5086-s005.zip › Immobilization/Chitosan(After swelling)/100X/IMG_3562.JPG]

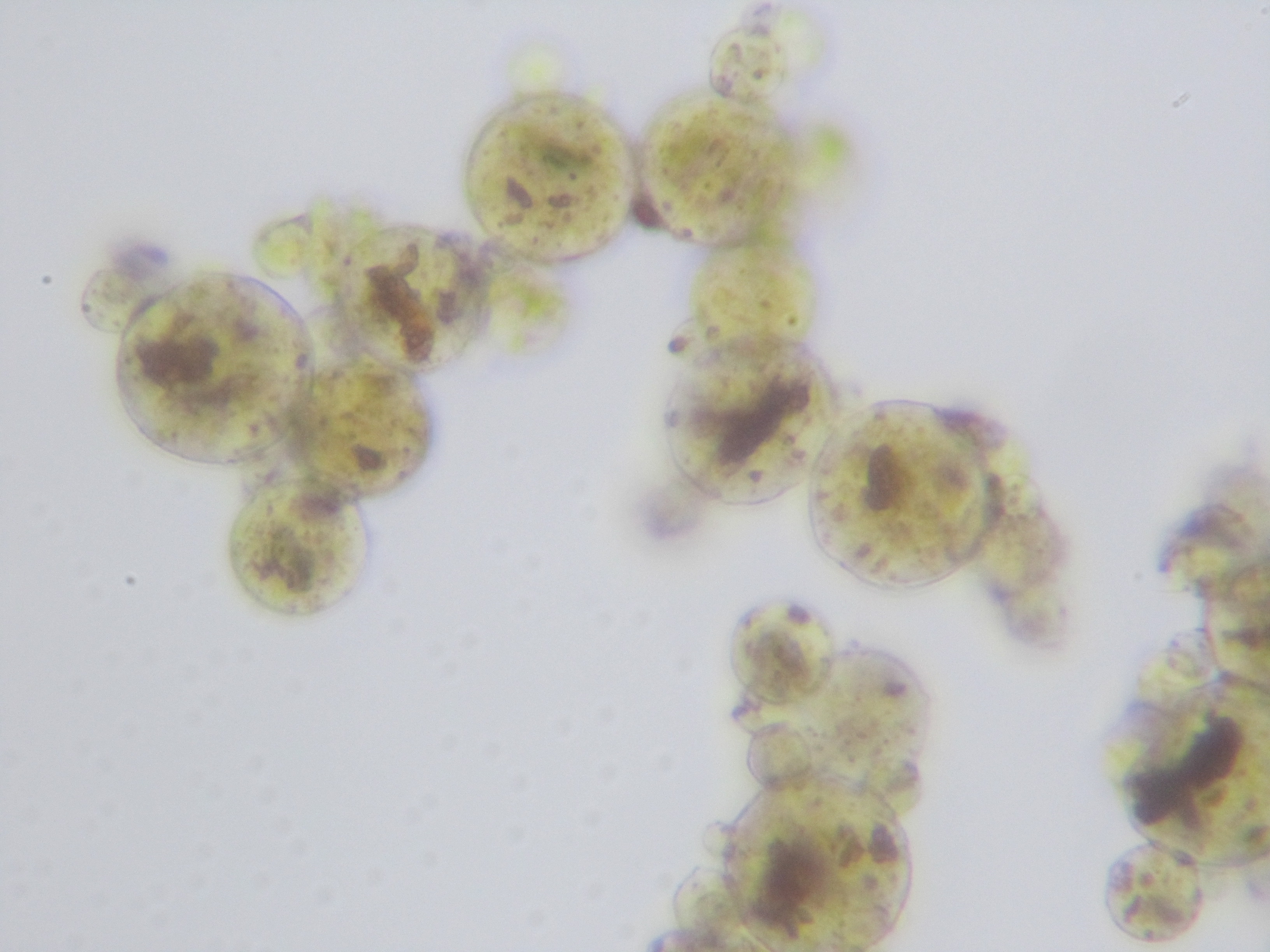

Supplement: Supplemental Information 5 [file peerj-06-5086-s005.zip › Immobilization/Chitosan(After swelling)/40X/IMG_3551.JPG]

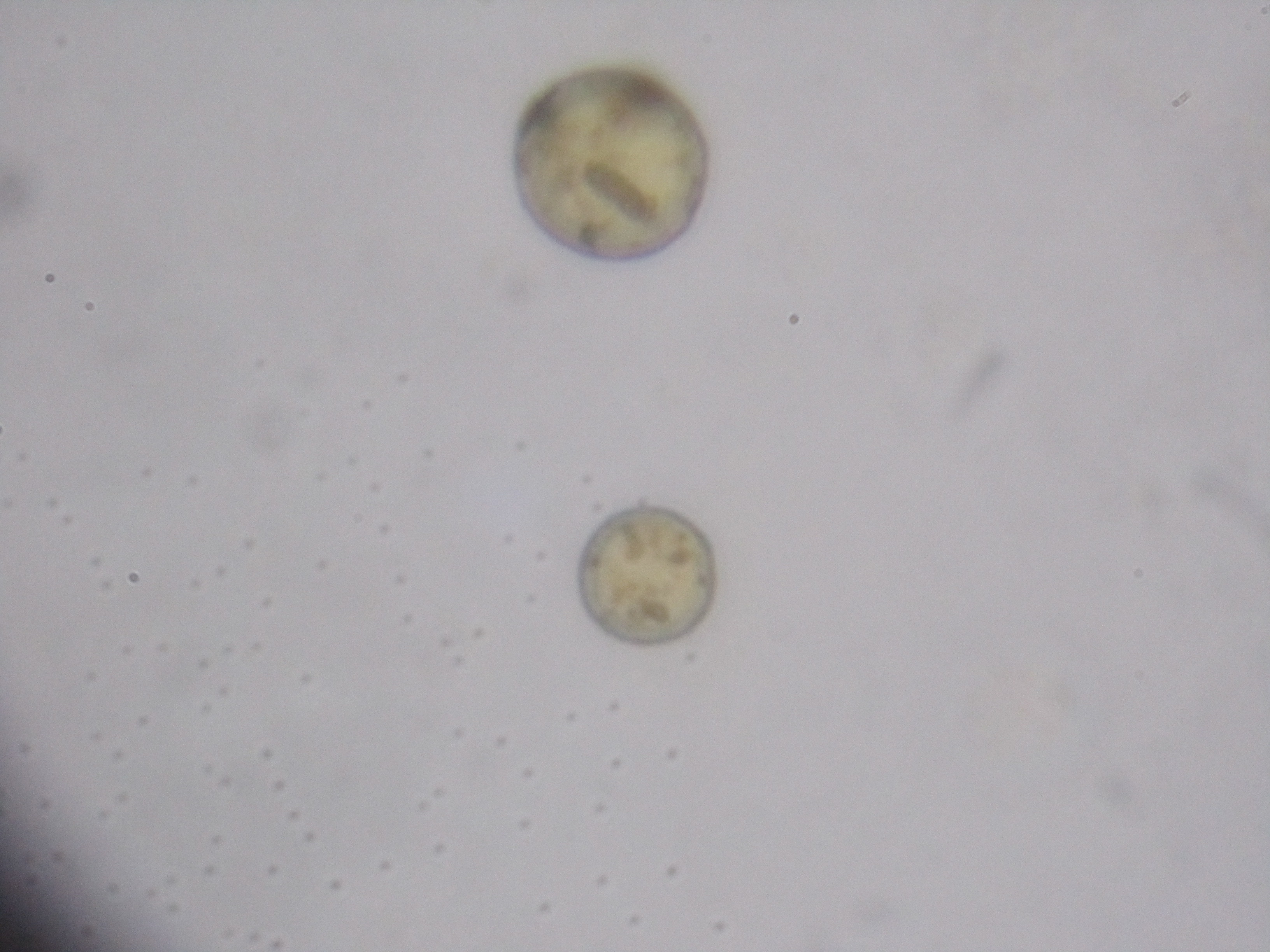

Supplement: Supplemental Information 5 [file peerj-06-5086-s005.zip › Immobilization/Chitosan(Before dryness)/100X/IMG_3591.JPG]

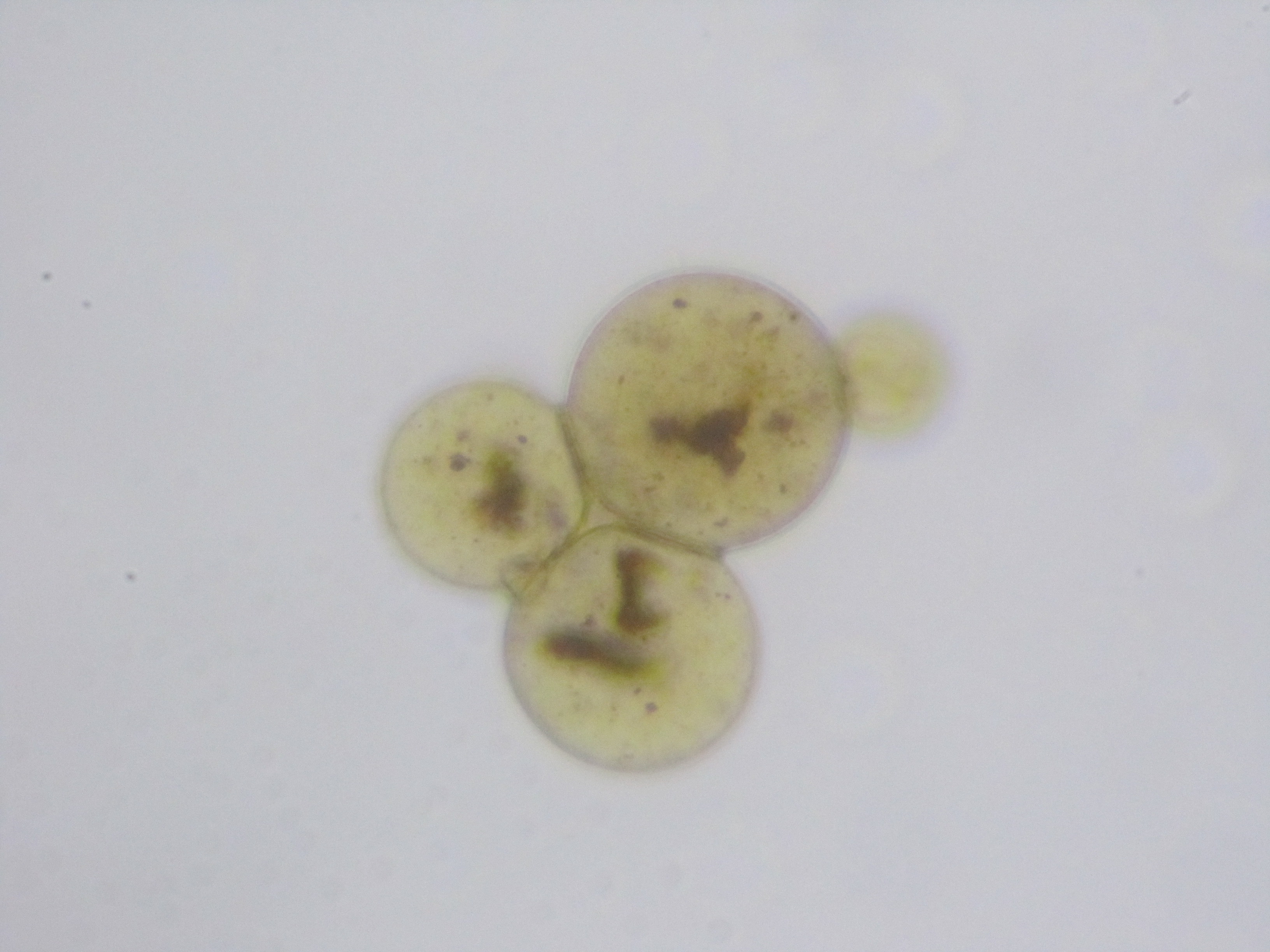

Supplement: Supplemental Information 5 [file peerj-06-5086-s005.zip › Immobilization/Chitosan(Before dryness)/40X/IMG_3575.JPG]

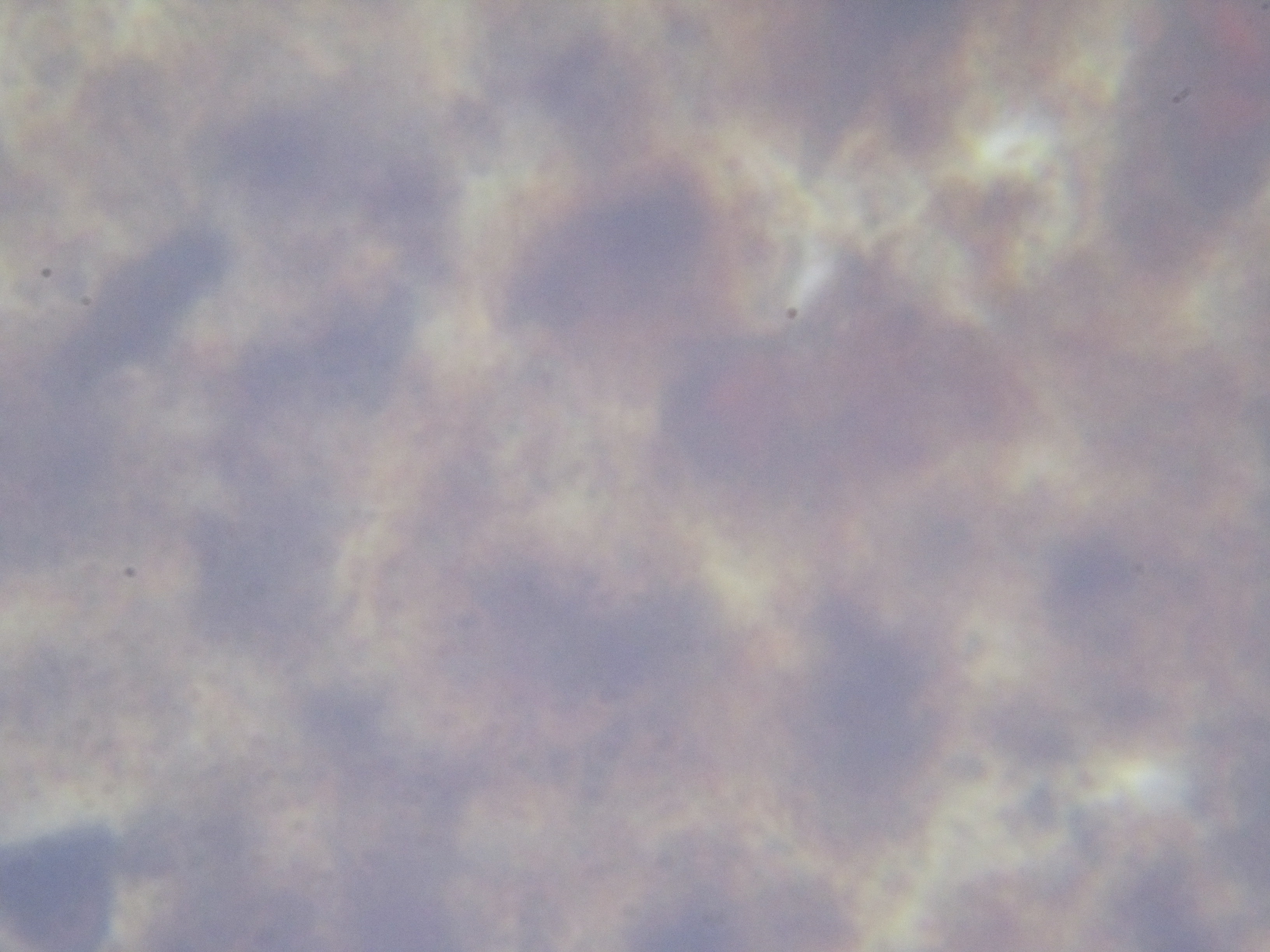

Supplement: Supplemental Information 5 [file peerj-06-5086-s005.zip › Immobilization/Chitosan(dryness)/IMG_3539.JPG]

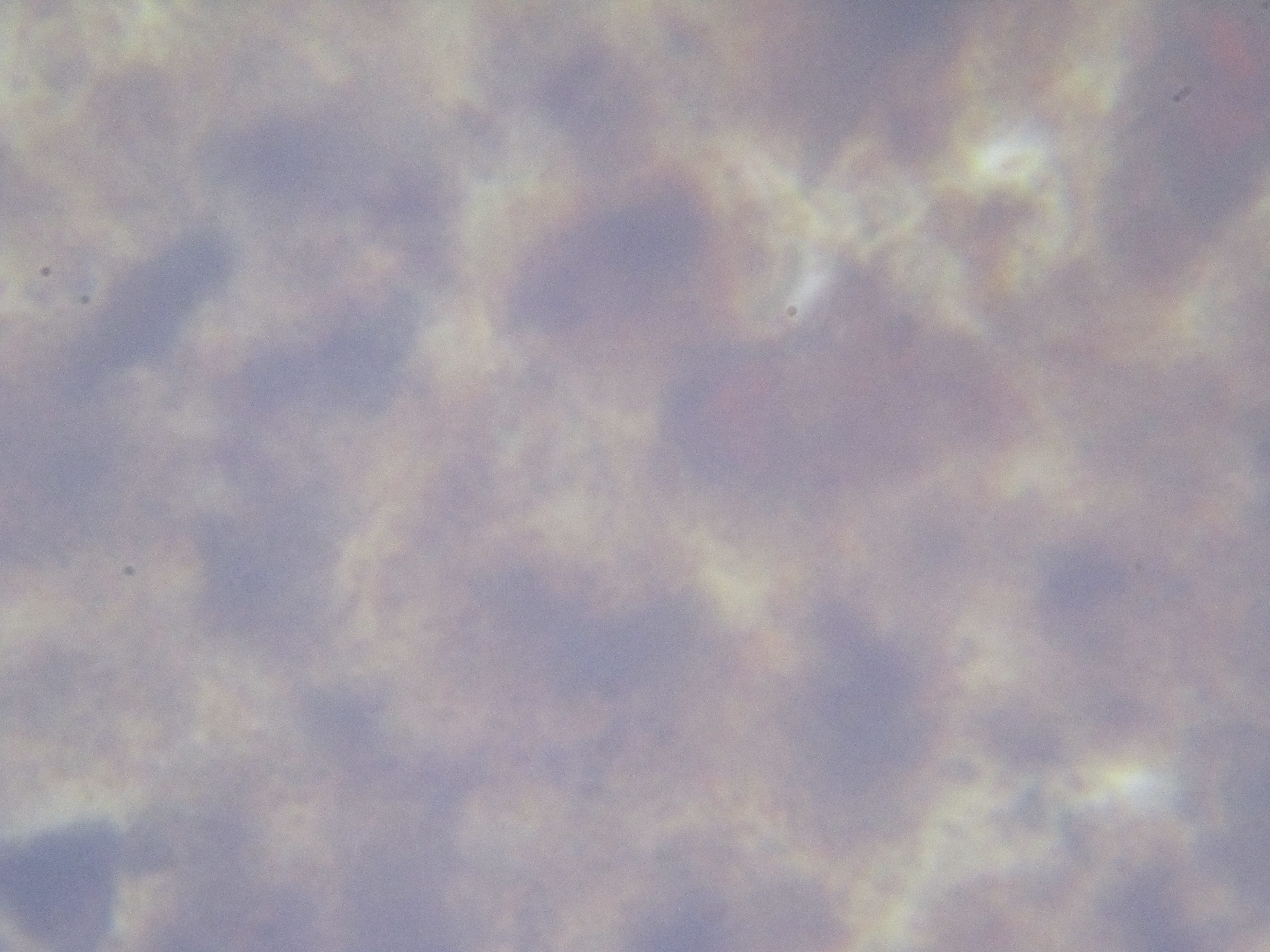

Supplement: Supplemental Information 5 [file peerj-06-5086-s005.zip › Immobilization/Chitosan(dryness)/IMG_3540.JPG]

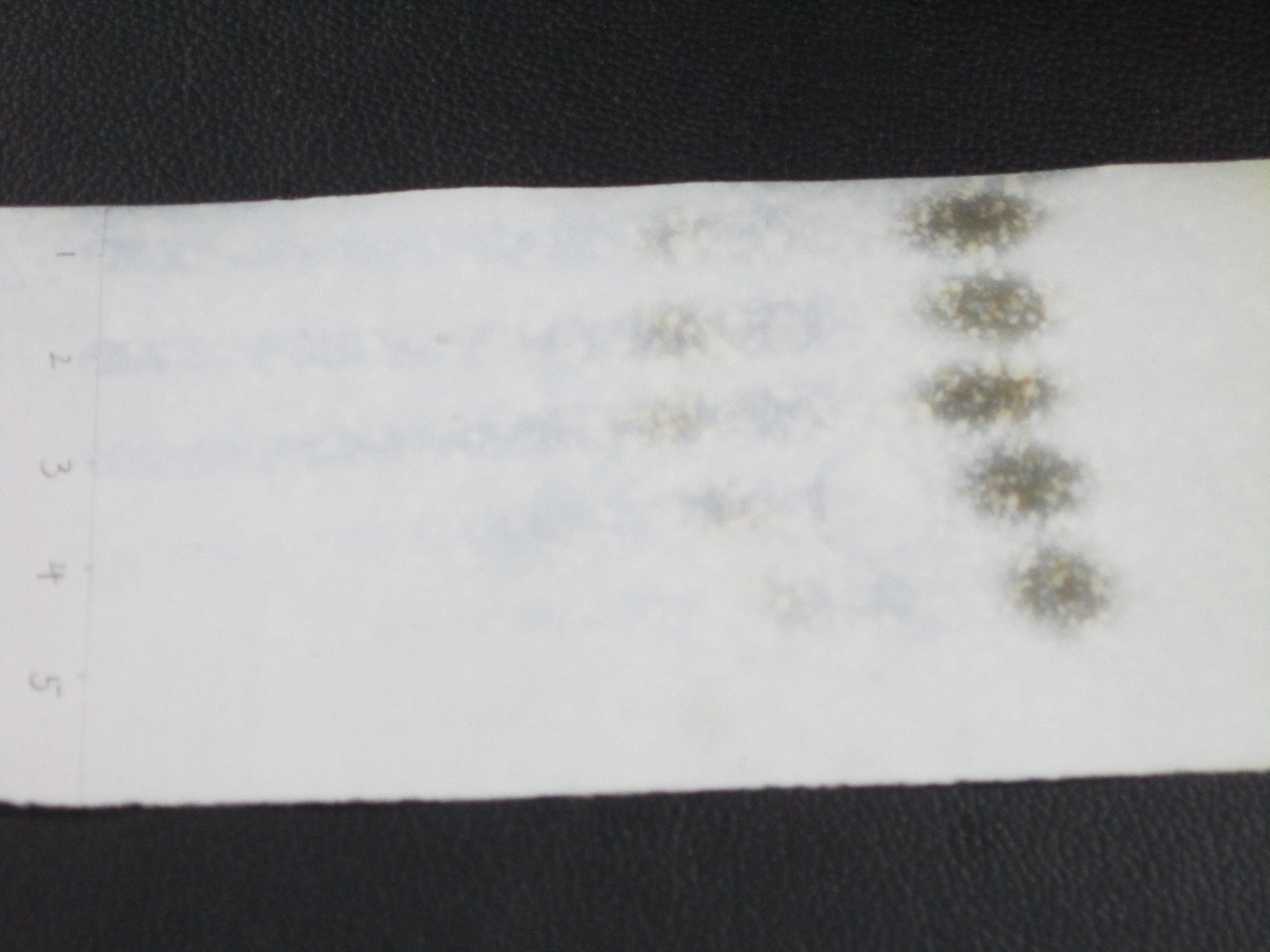

Supplement: Supplemental Information 6 [file peerj-06-5086-s006.zip › TLC/07-TLC/TLC1.JPG]

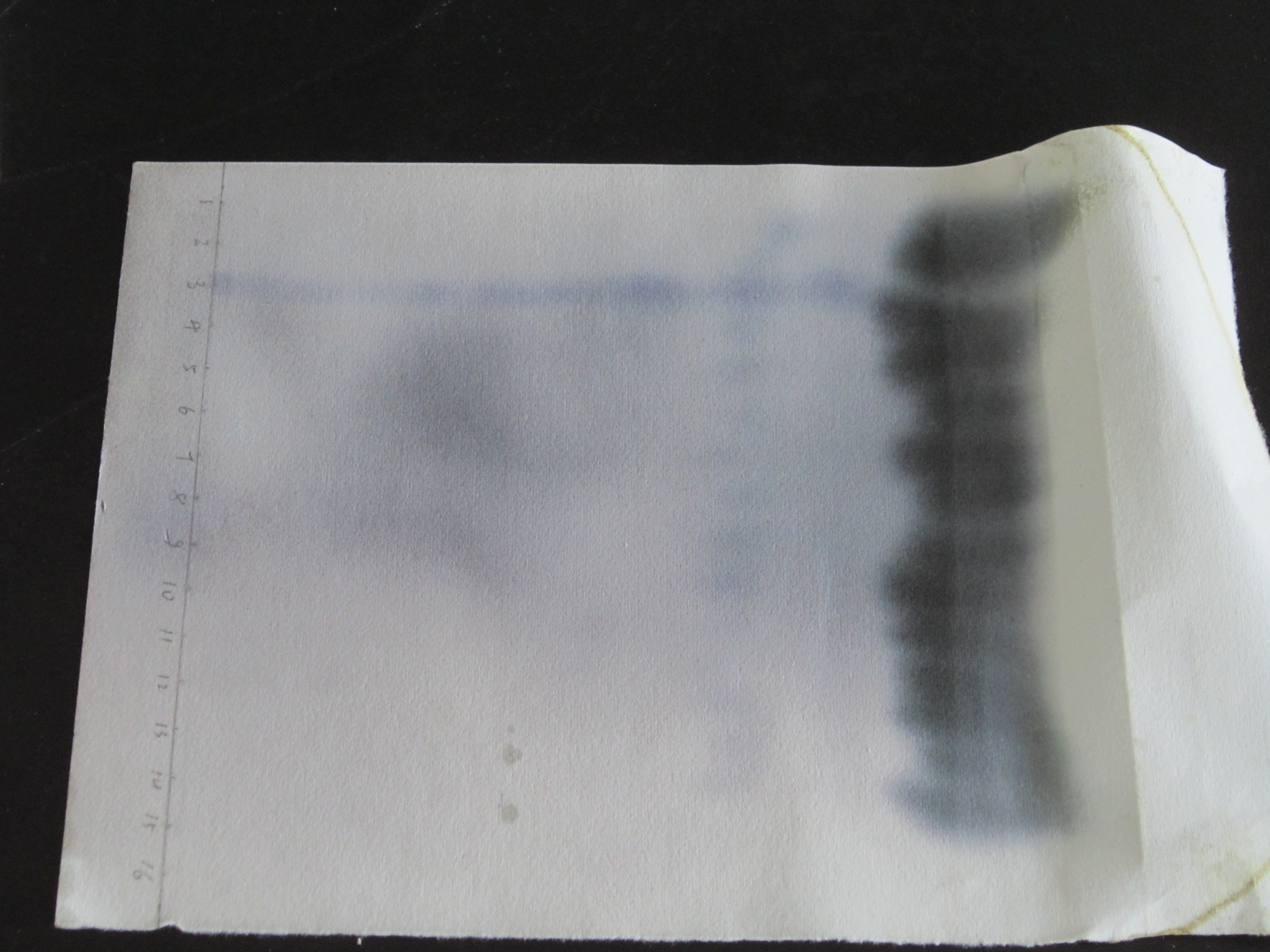

Supplement: Supplemental Information 6 [file peerj-06-5086-s006.zip › TLC/07-TLC/TLC2.JPG]

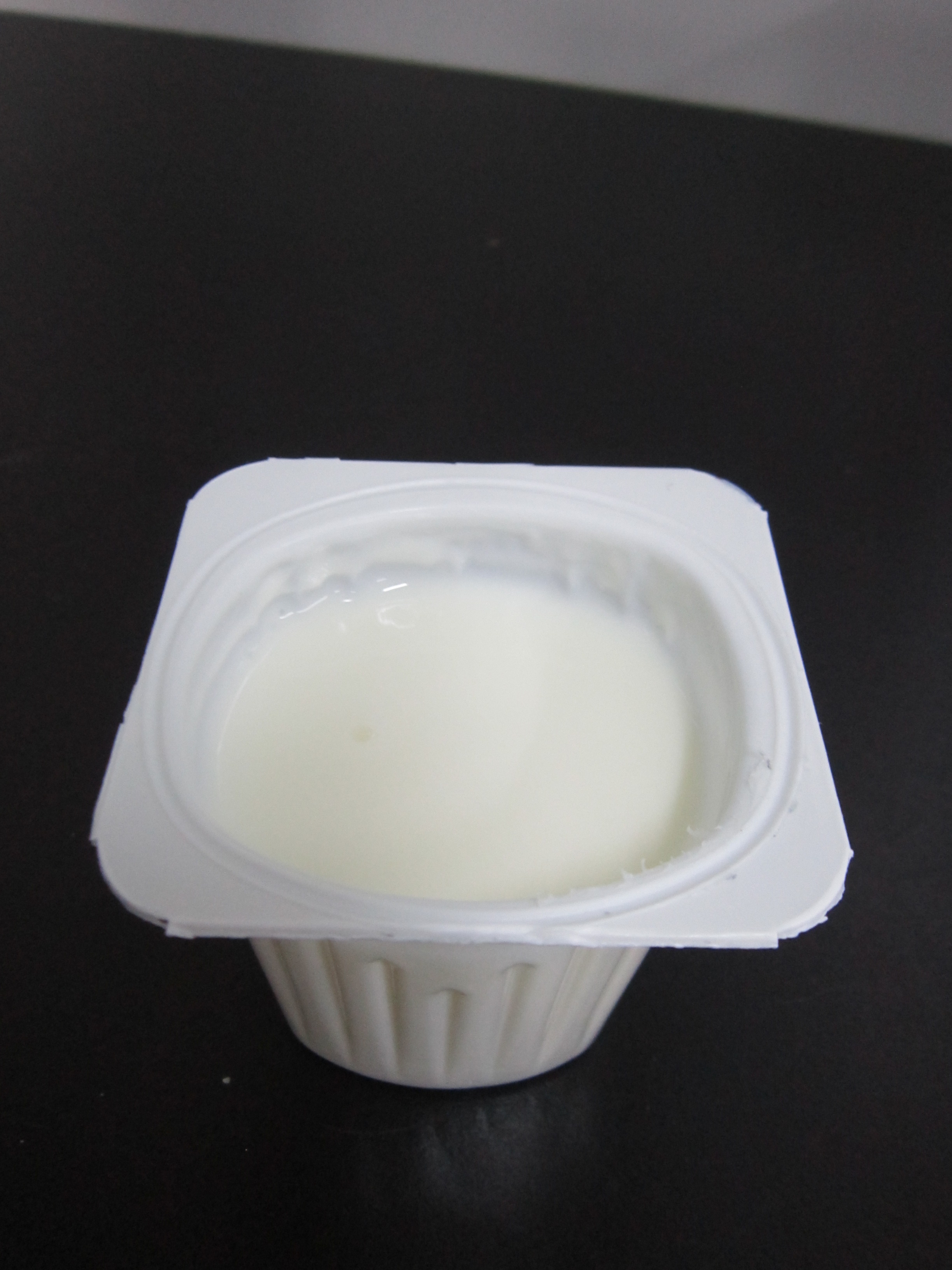

Supplement: Supplemental Information 8 [file peerj-06-5086-s008.zip › IMO application/IMO Yoghourt/01Yoghourt.JPG]

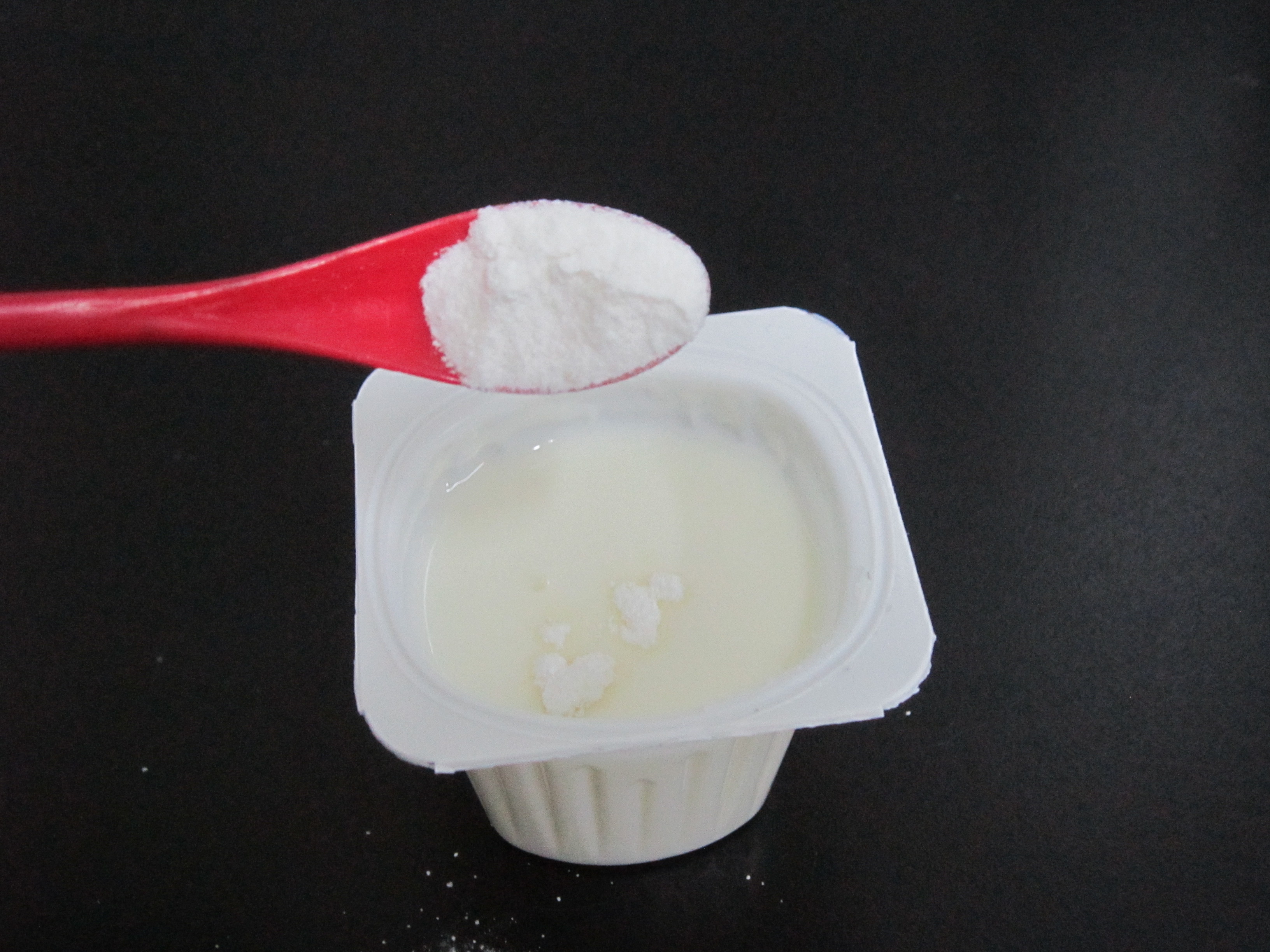

Supplement: Supplemental Information 8 [file peerj-06-5086-s008.zip › IMO application/IMO Yoghourt/02-added IMO 1.JPG]

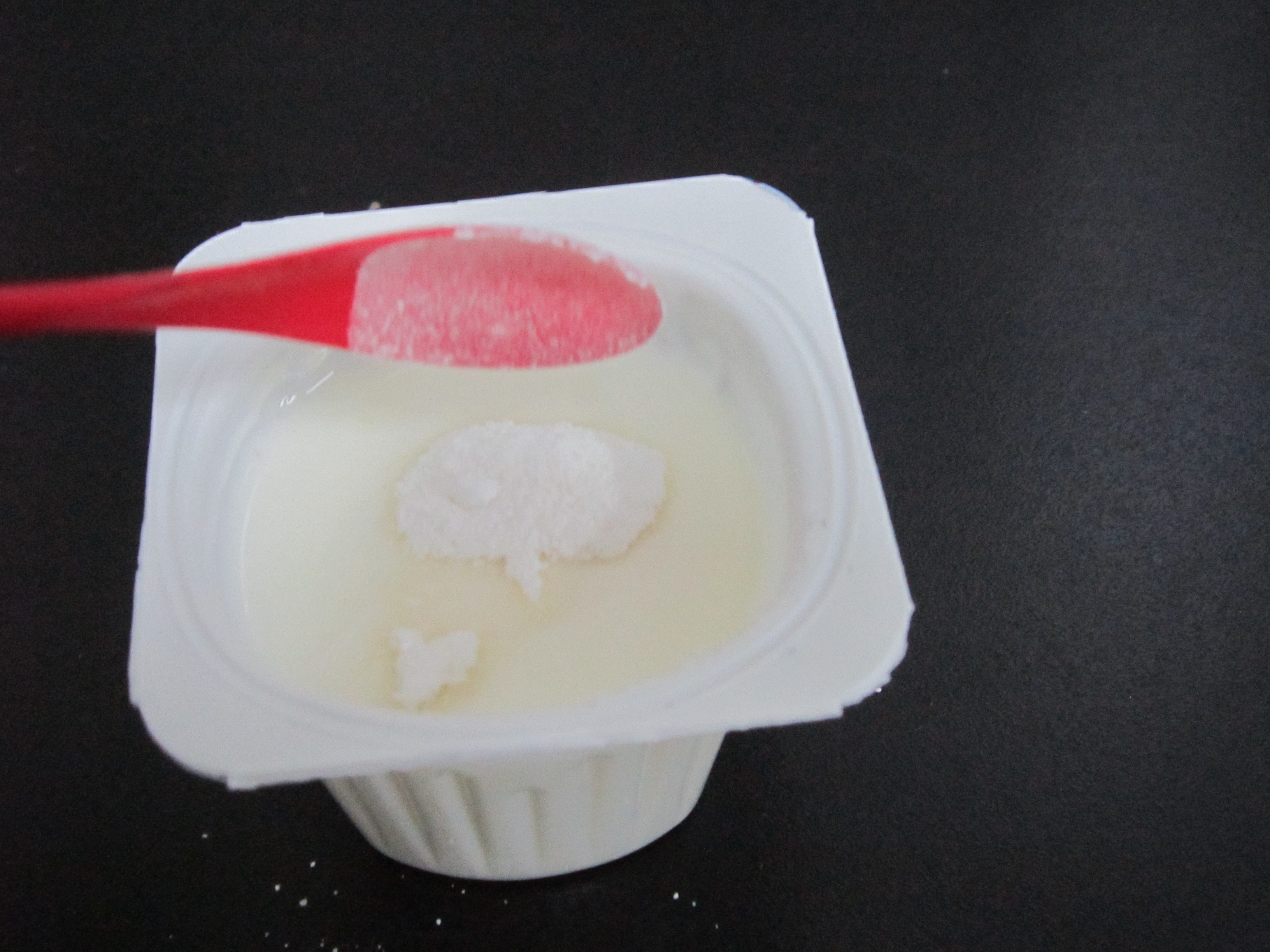

Supplement: Supplemental Information 8 [file peerj-06-5086-s008.zip › IMO application/IMO Yoghourt/03-added IMO 2.JPG]

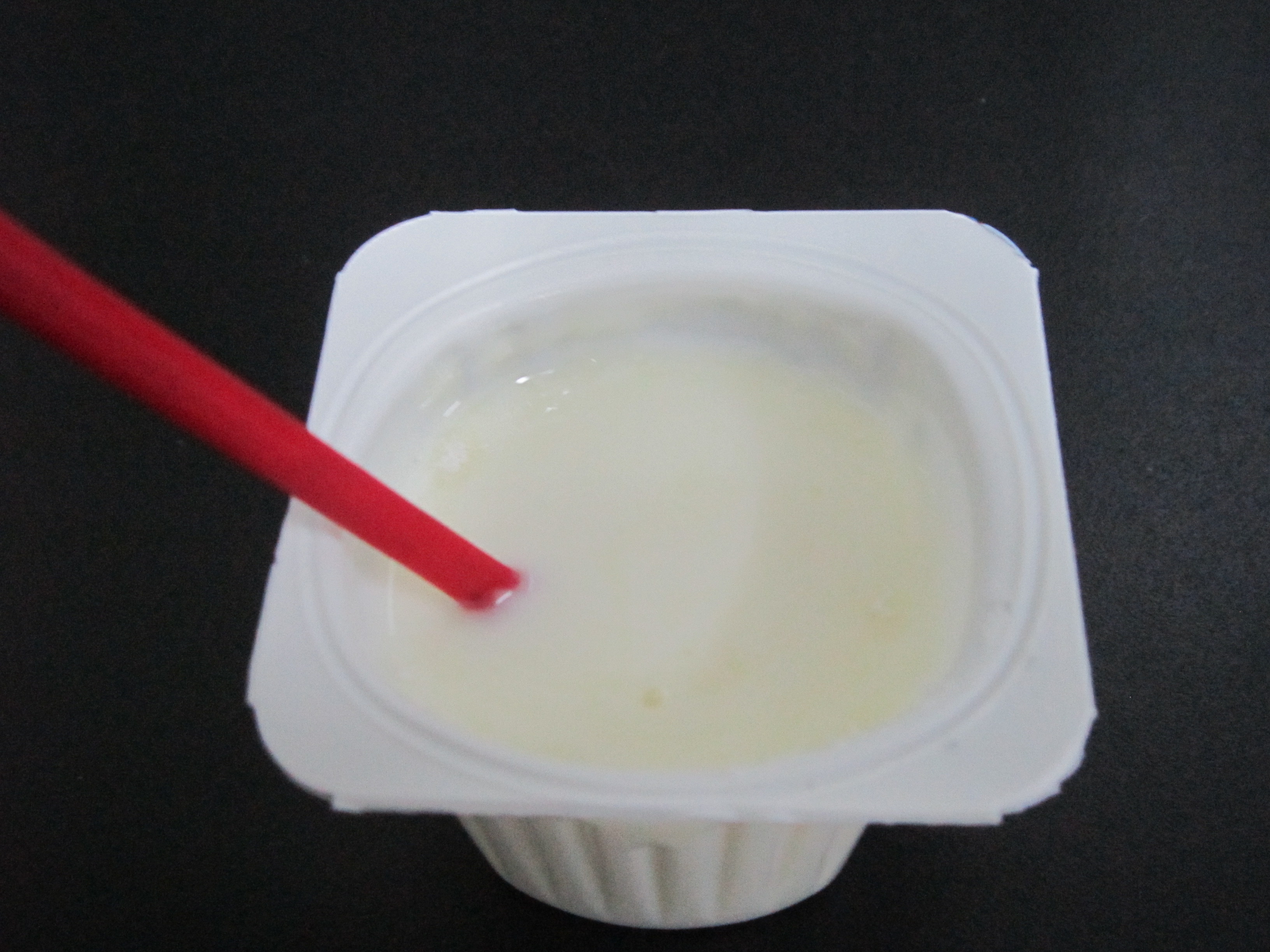

Supplement: Supplemental Information 8 [file peerj-06-5086-s008.zip › IMO application/IMO Yoghourt/04-Mixed.JPG]

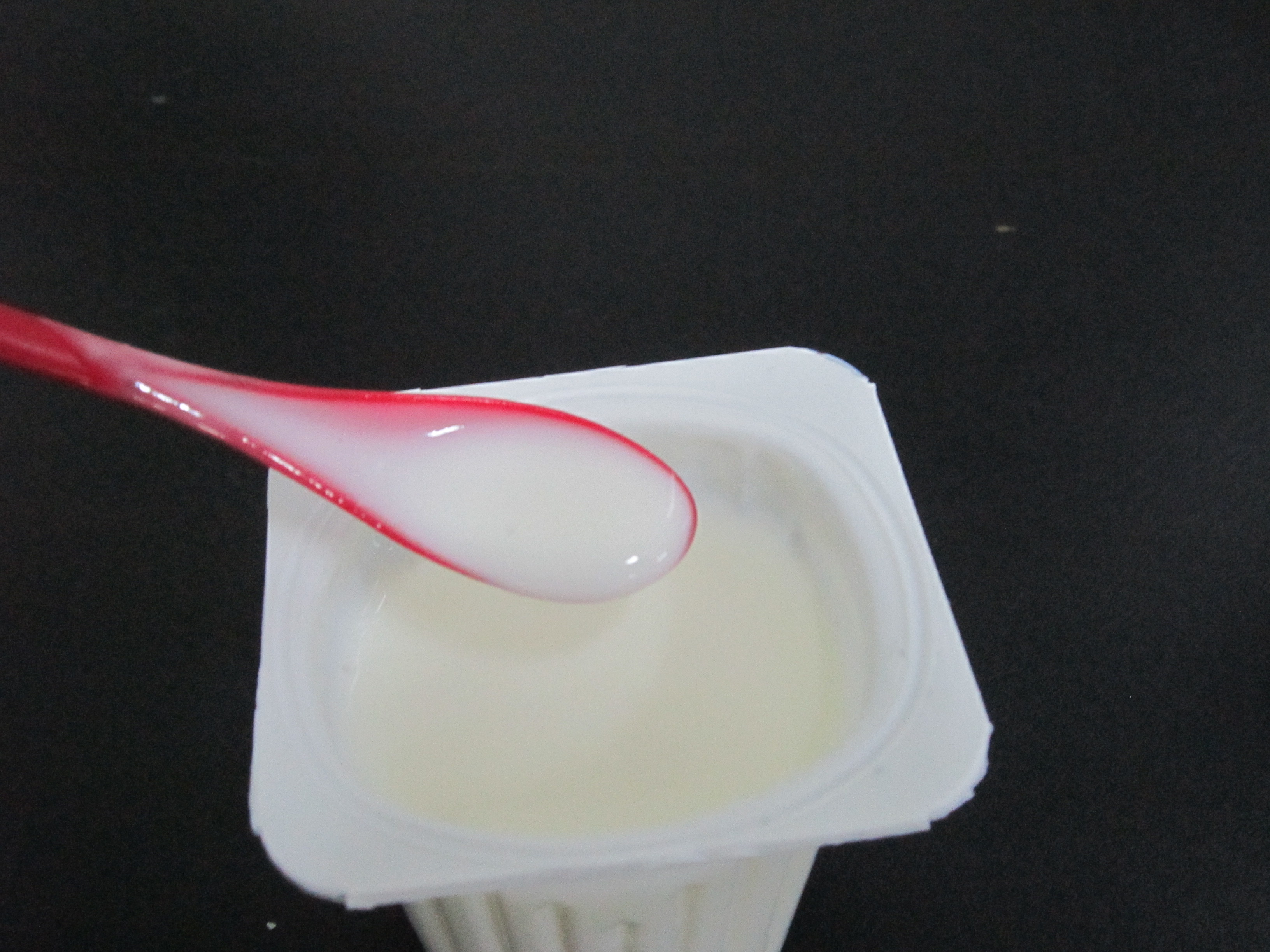

Supplement: Supplemental Information 8 [file peerj-06-5086-s008.zip › IMO application/IMO Yoghourt/05-IMO Yoghourt 01.JPG]

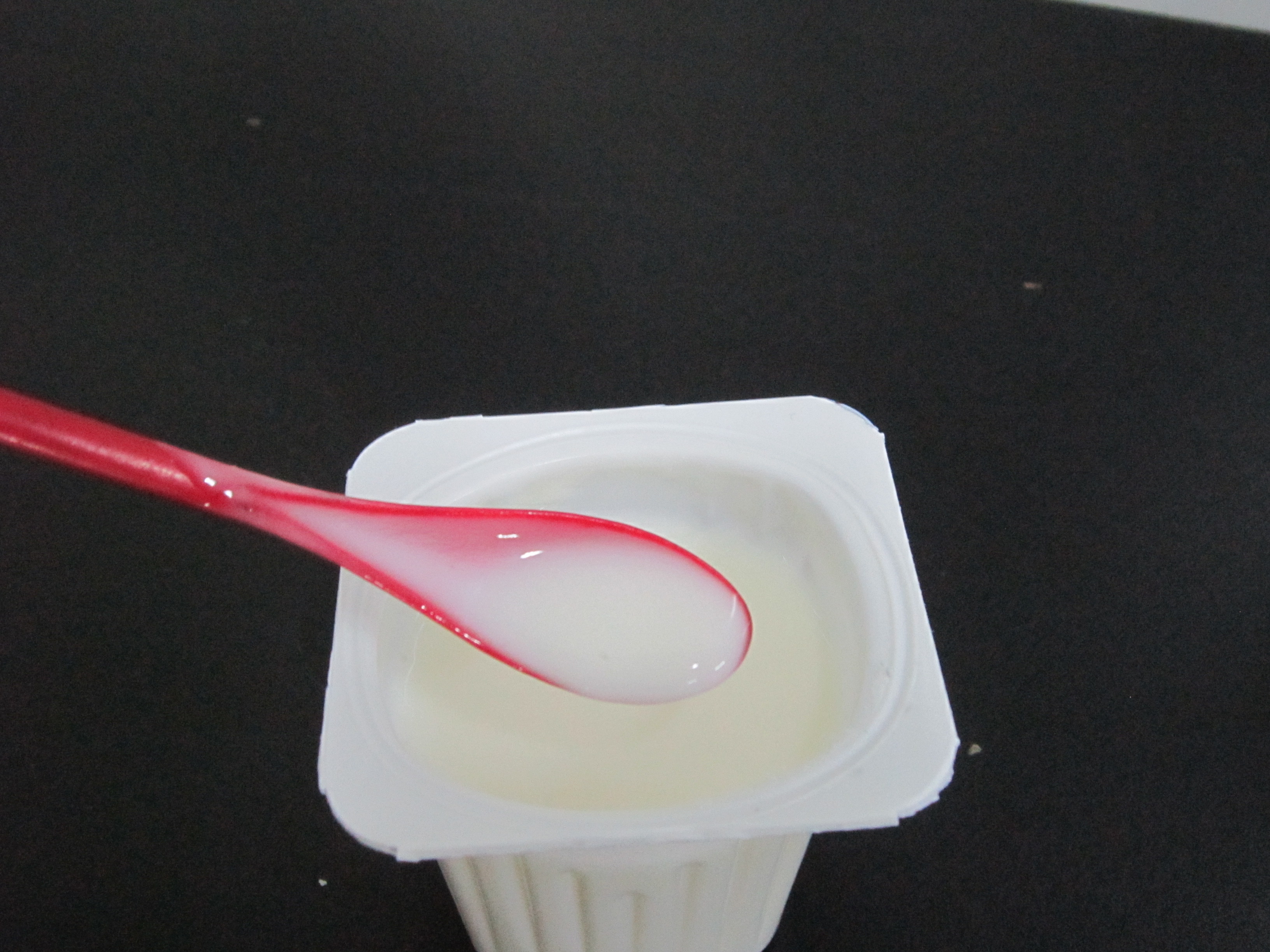

Supplement: Supplemental Information 8 [file peerj-06-5086-s008.zip › IMO application/IMO Yoghourt/05-IMO Yoghourt 02.JPG]

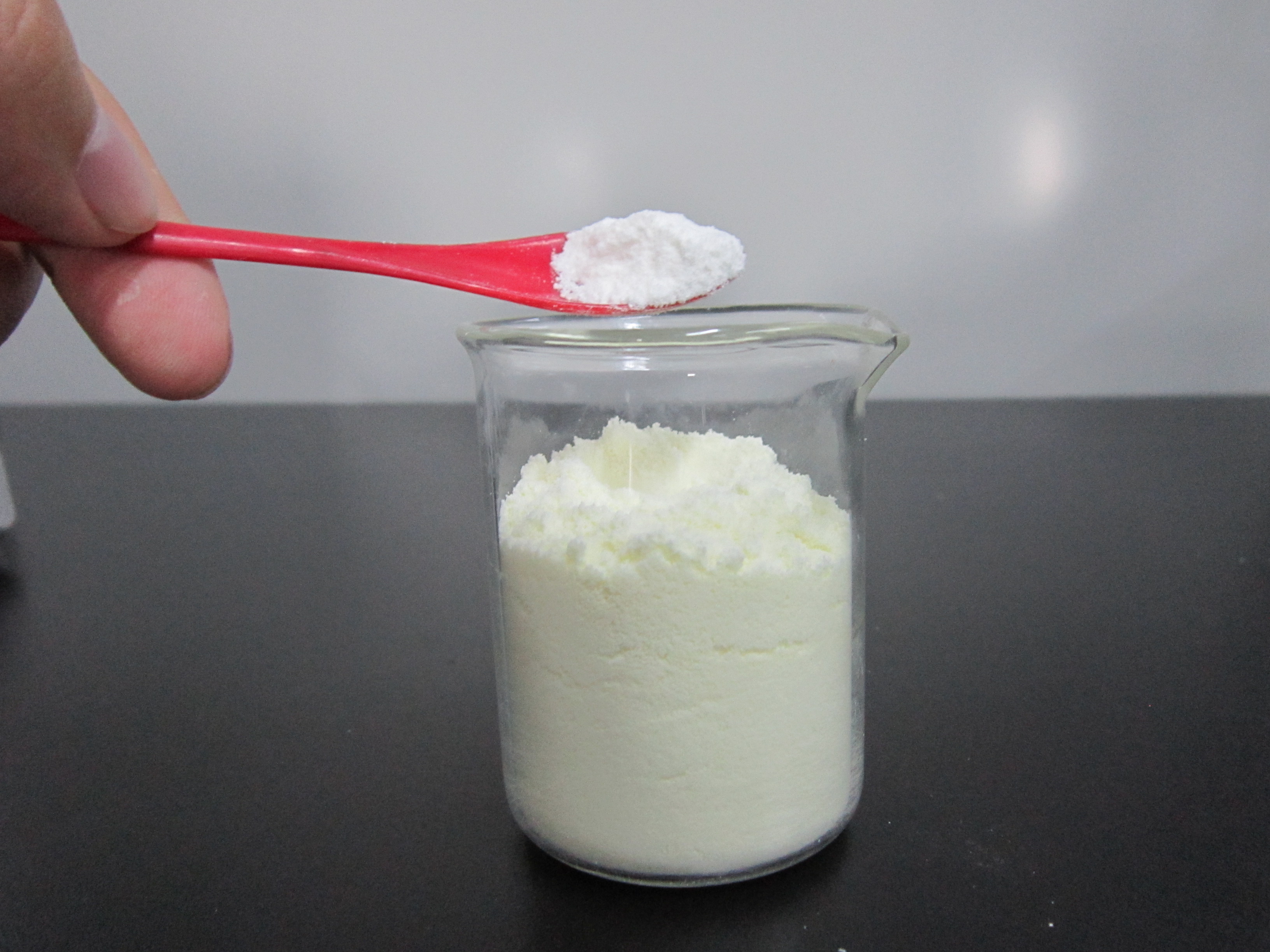

Supplement: Supplemental Information 8 [file peerj-06-5086-s008.zip › IMO application/IMO milk powder/Added IMO 01.JPG]

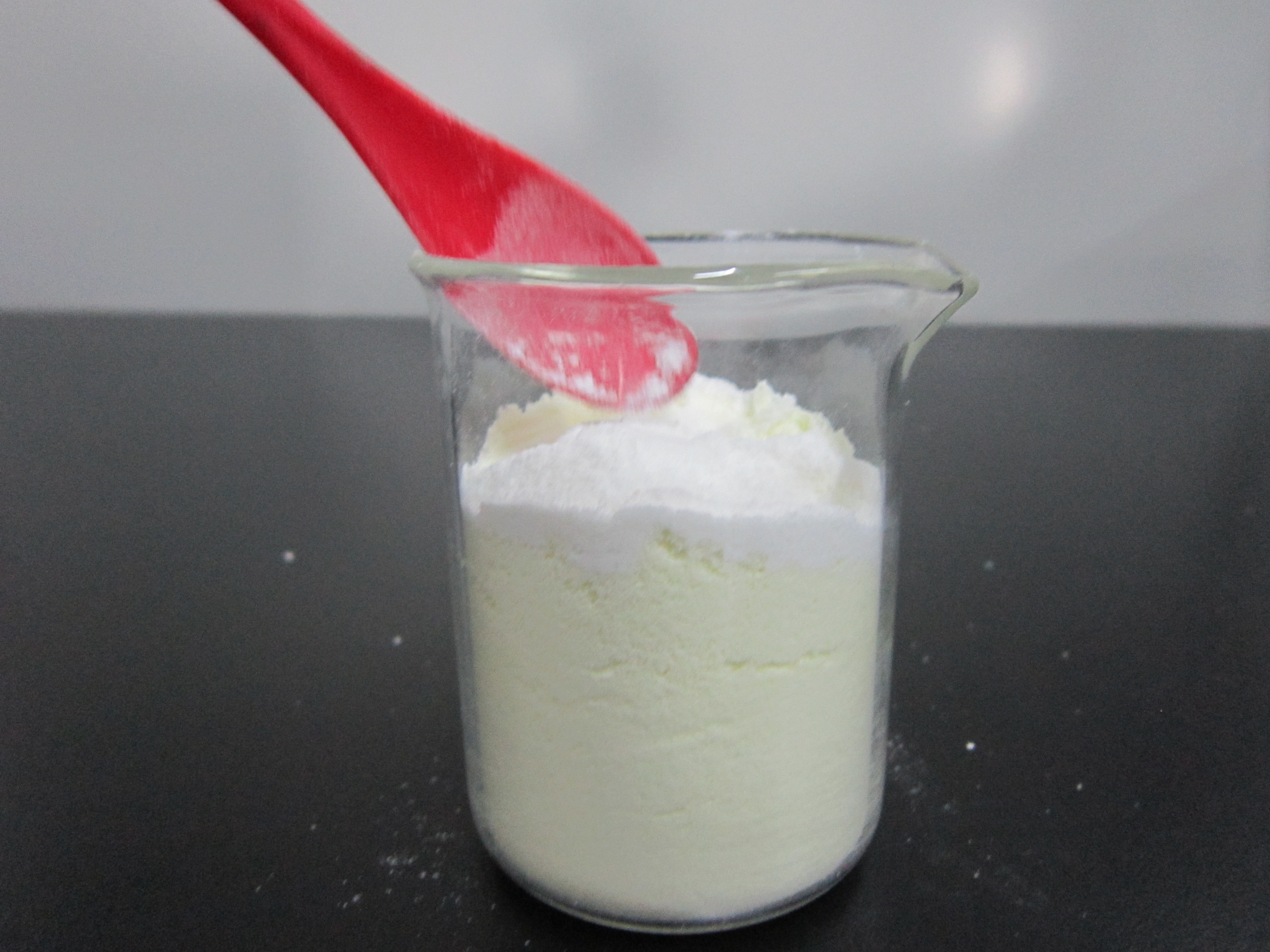

Supplement: Supplemental Information 8 [file peerj-06-5086-s008.zip › IMO application/IMO milk powder/Added IMO 02.JPG]

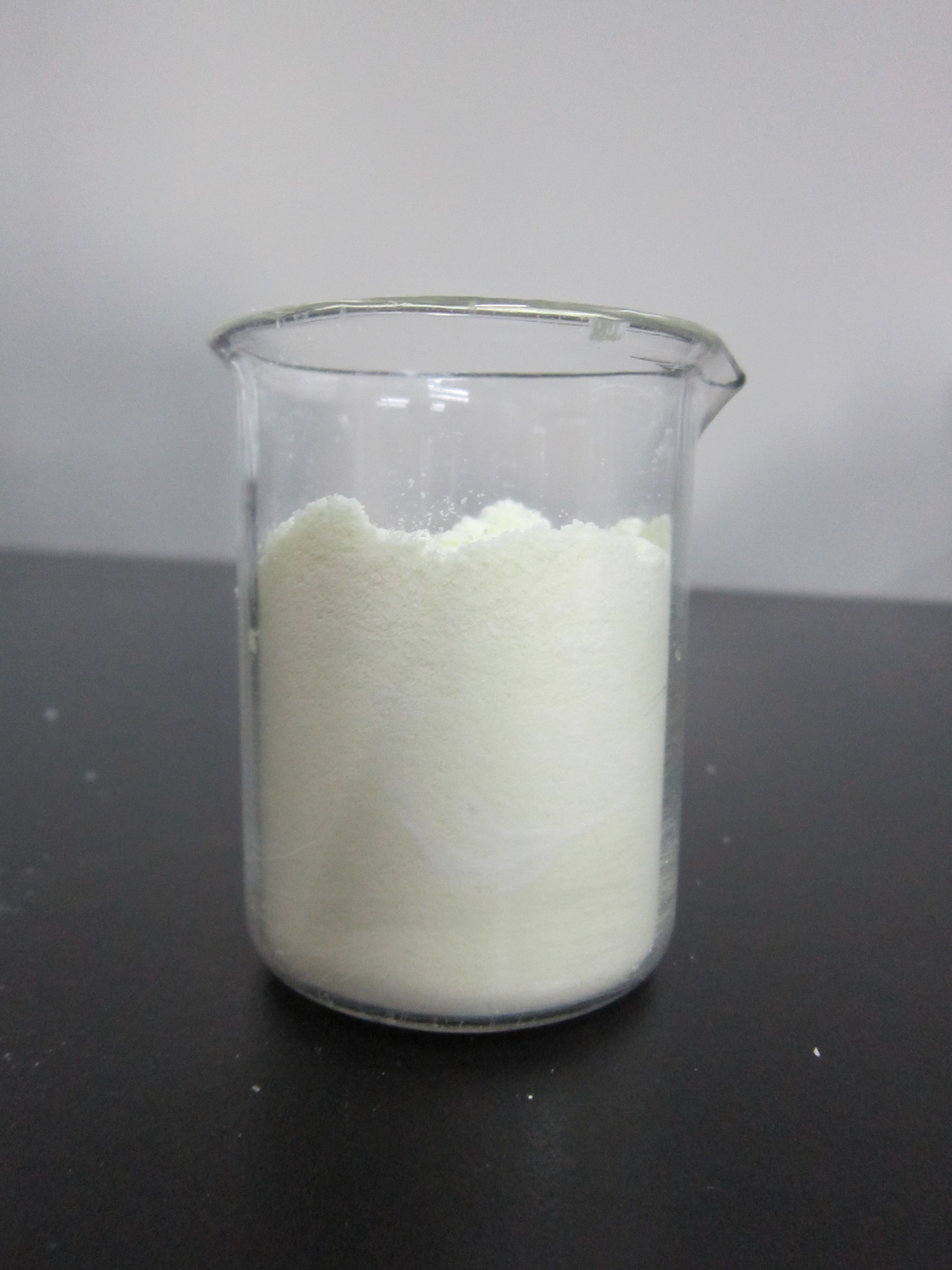

Supplement: Supplemental Information 8 [file peerj-06-5086-s008.zip › IMO application/IMO milk powder/IMO milk powder.JPG]

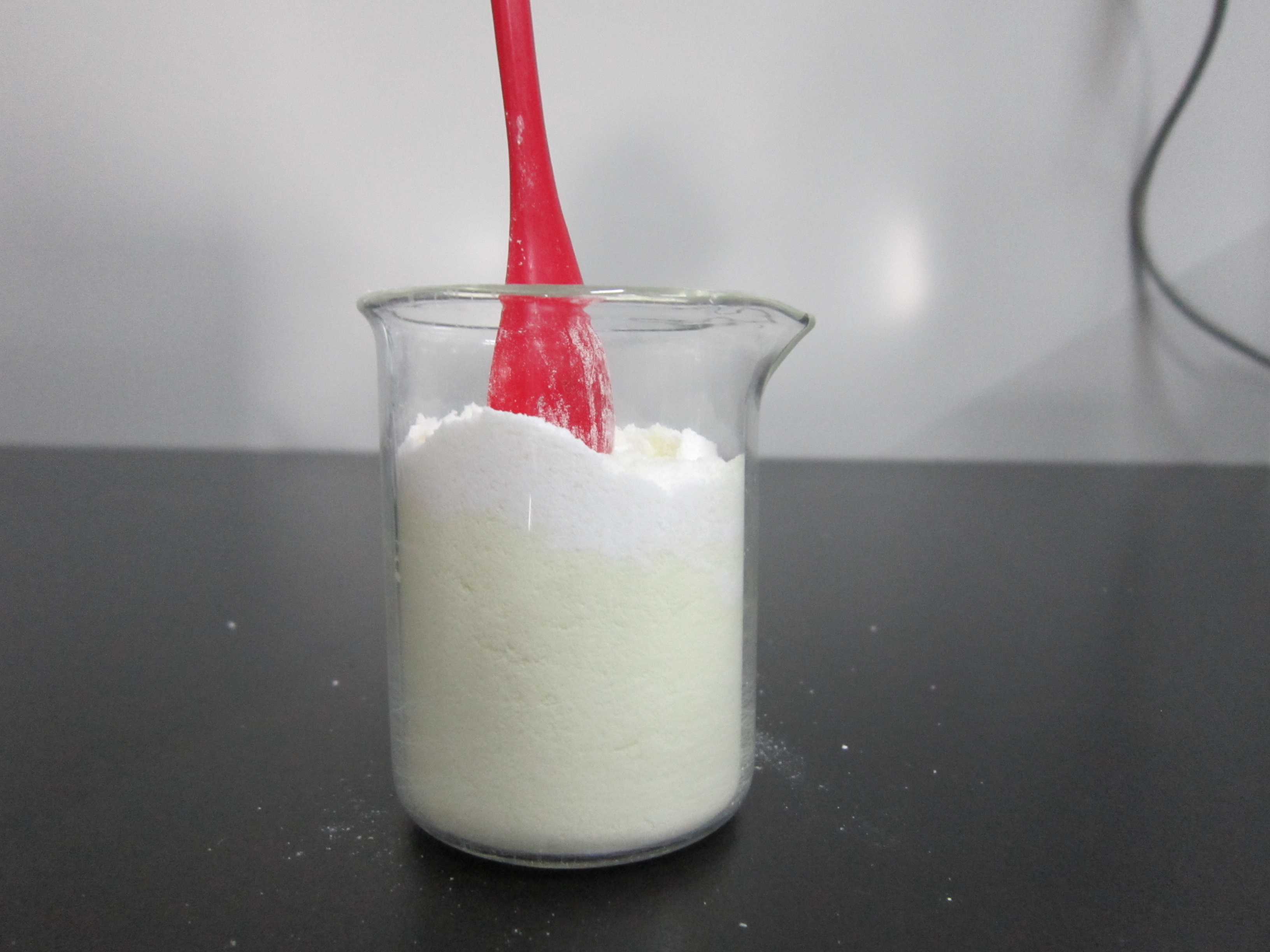

Supplement: Supplemental Information 8 [file peerj-06-5086-s008.zip › IMO application/IMO milk powder/Mixed.JPG]

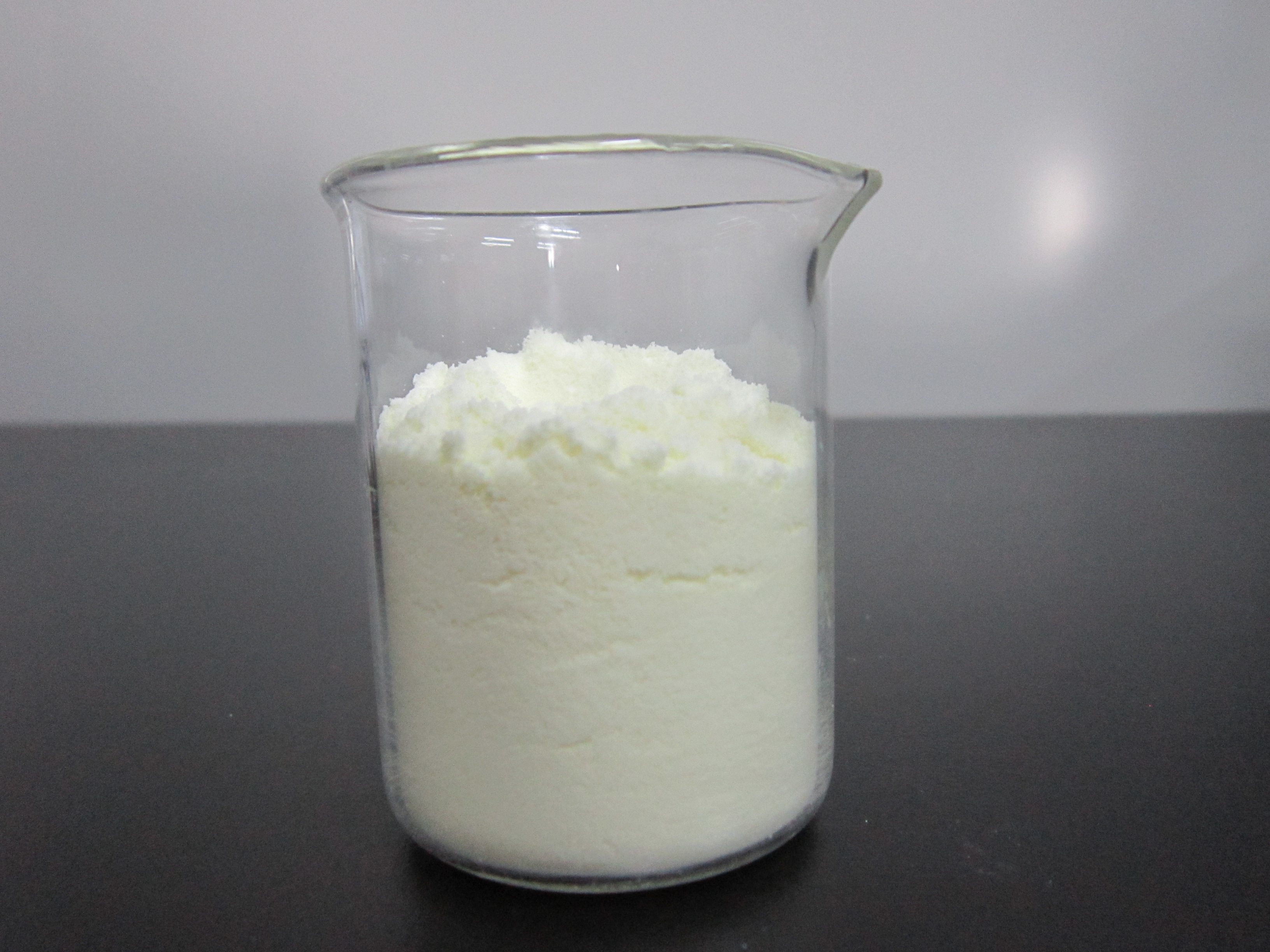

Supplement: Supplemental Information 8 [file peerj-06-5086-s008.zip › IMO application/IMO milk powder/milk powder.JPG]
