# Supplementary material for: Starch biotransformation into isomaltooligosaccharides using thermostable alpha-glucosidase from Geobacillus stearothermophilus
Source: PeerJ. 2018 Jun 21;6:e5086. doi: 10.7717/peerj.5086 (PMC6015754; doi:10.7717/peerj.5086)
Supplement: Supplemental Information 8 [file peerj-06-5086-s008.zip › IMO application/Process flow.pptx]

## Slide 1
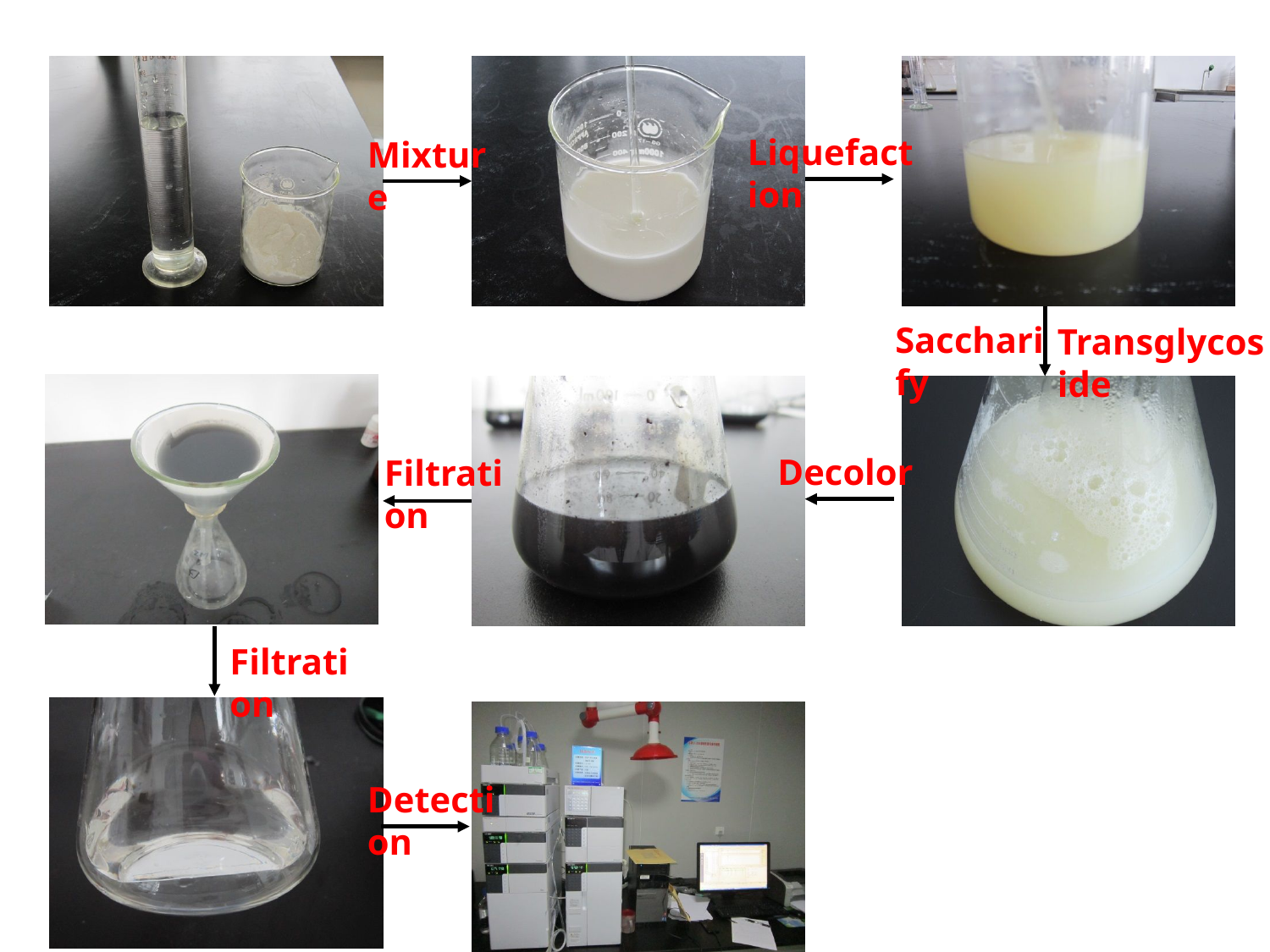

Liquefaction
Mixture
Saccharify
Transglycoside
Decolor
Filtration
Filtration
Detection
