# Supplementary material for: Starch biotransformation into isomaltooligosaccharides using thermostable alpha-glucosidase from Geobacillus stearothermophilus
Source: PeerJ. 2018 Jun 21;6:e5086. doi: 10.7717/peerj.5086 (PMC6015754; doi:10.7717/peerj.5086)
Supplement: Supplemental Information 7 [file peerj-06-5086-s007.zip › HPLC/HPLC-0404.doc]

仪器信息：

实验条件：

**IMO大量制备：第一批次(EAG-SKS)**

峰号 保留时间	面积	高度	面积%

1	2.428	2834048	228111	11.362

2	4.754	11069655	673134	44.378

3	4.884	9339895	676272	37.444

4	6.041	924551	44733	3.707

5	7.922	47721	3169	0.191

6	8.764	372612	19542	1.494

7	11.964	242141	9660	0.971

8	16.391	113203	2306	0.454

**IMO对照：山东保龄宝(10%, w/v)**

峰号	保留时间	面积	高度	面积%

1	2.416	4111763	326102	0.000	40.022

2	3.405	142129	15248	0.000	1.383

3	6.070	756033	31911	0.000	7.359

4	6.584	1911189	76830	0.000	18.603

5	8.042	101134	3825	0.000	0.984

6	8.923	1929528	56588	0.000	18.781

7	9.705	418998	17174	0.000	4.078

8	12.066	304480	10168	0.000	2.964

9	13.233	303852	10587	0.000	2.958

10	16.511	124525	3108	0.000	1.212

11	17.933	170162	3316	0.000	1.656
