# Supplementary material for: Starch biotransformation into isomaltooligosaccharides using thermostable alpha-glucosidase from Geobacillus stearothermophilus
Source: PeerJ. 2018 Jun 21;6:e5086. doi: 10.7717/peerj.5086 (PMC6015754; doi:10.7717/peerj.5086)
Supplement: Supplemental Information 7 [file peerj-06-5086-s007.zip › HPLC/HPLC-0409.doc]

SKS PILIANG

峰号 保留时间	面积	高度	面积%

1	2.428	2834048	228111	11.362

2	4.754	11069655	673134	44.378

3	4.884	9339895	676272	37.444

4	6.041	924551	44733	3.707

5	7.922	47721	3169	0.191

6	8.764	372612	19542	1.494

7	11.964	242141	9660	0.971

8	16.391	113203	2306	0.454

BYB IMO

峰号	化合物名	保留时间	面积	高度	标记	浓度	浓度单位	化合物ID号	面积%

1	2.416	4111763	326102	S	0.000	40.022

2	3.405	142129	15248	T	0.000	1.383

3	6.070	756033	31911	0.000	7.359

4	6.584	1911189	76830	V	0.000	18.603

5	8.042	101134	3825	0.000	0.984

6	8.923	1929528	56588	V	0.000	18.781

7	9.705	418998	17174	V	0.000	4.078

8	12.066	304480	10168	0.000	2.964

9	13.233	303852	10587	M	0.000	2.958

10	16.511	124525	3108	M	0.000	1.212

11	17.933	170162	3316	M	0.000	1.656

RS-IMO

峰号	化合物名	保留时间	面积	高度	标记	浓度	浓度单位	化合物ID号	面积%

1	2.416	3328014	257219	0.000	36.623

2	3.404	426661	38570	V	0.000	4.695

3	B2	4.541	312188	22681	0.406	mg/L	1	3.435

4	5.875	1083664	46037	0.000	11.925

5	6.594	2912712	128461	V	0.000	32.053

6	7.991	214154	11622	0.000	2.357

7	9.520	326780	14201	0.000	3.596

8	10.881	132017	6081	0.000	1.453

9	13.908	67525	2224	M	0.000	0.743

10	14.965	111907	3727	M	0.000	1.231

11	20.461	171582	3891	M	0.000	1.888

总计	9087204	534714	0.406	100.000

SH

峰号	化合物名	保留时间	面积	高度	标记	浓度	浓度单位	化合物ID号	面积%

1	2.419	2873729	225698	0.000	16.485

2	3.413	269234	23556	V	0.000	1.544

3	B2	4.638	10699238	582532	13.906	mg/L	1	61.376

4	5.881	962961	38179	V	0.000	5.524

5	6.576	2126897	105505	V	0.000	12.201

6	7.977	129167	8240	0.000	0.741

7	9.484	184393	9393	0.000	1.058

8	10.843	57604	3233	M	0.000	0.330

9	14.930	129163	3694	M	0.000	0.741

总计	17432385	1000032	13.906	100.000

SKS-2

峰号	化合物名	保留时间	面积	高度	标记	浓度	浓度单位	化合物ID号	面积%

1	2.427	2061823	186093	0.000	8.056

2	2.823	182593	10250	V	0.000	0.713

3	B2	4.742	11634826	685926	15.122	mg/L	1	45.459

4	4.876	10152956	680455	V	0.000	39.669

5	5.957	995465	38205	V	0.000	3.889

6	6.475	177537	11457	V	0.000	0.694

7	7.834	34005	2323	M	0.000	0.133

8	8.615	252772	14302	0.000	0.988

9	11.691	101885	5082	0.000	0.398

总计	25593863	1634093	15.122	100.000

SKS-1

峰号	化合物名	保留时间	面积	高度	标记	浓度	浓度单位	化合物ID号	面积%

1	2.427	2256929	185016	S	0.000	8.688

2	B2	4.767	22487466	699972	29.227	mg/L	1	86.561

3	5.955	765195	30391	V	0.000	2.945

4	6.486	205237	13921	V	0.000	0.790

5	7.811	15041	1259	M	0.000	0.058

6	8.609	161875	9612	0.000	0.623

7	11.680	86946	3901	M	0.000	0.335

总计	25978689	944073	29.227	100.000

TY

峰号	化合物名	保留时间	面积	高度	标记	浓度	浓度单位	化合物ID号	面积%

1	2.421	2409177	208208	0.000	11.296

2	2.850	249590	12546	V	0.000	1.170

3	3.416	111332	8407	V	0.000	0.522

4	B2	4.693	15800399	644322	20.536	mg/L	1	74.087

5	5.966	700857	23571	V	0.000	3.286

6	6.534	1161303	64634	V	0.000	5.445

7	7.918	22543	1449	M	0.000	0.106

8	8.681	501994	23924	0.000	2.354

9	9.483	93888	5782	M	0.000	0.440

10	11.707	114800	4880	M	0.000	0.538

11	12.782	161073	5501	M	0.000	0.755

总计	21326957	1003224	20.536	100.000
